# Perfectionism, Self-Esteem, and the Will to Win Among Adolescent Athletes: The Effects of the Level of Achievements and Gender

**DOI:** 10.3389/fpsyg.2021.580446

**Published:** 2021-08-10

**Authors:** Md. Dilsad Ahmed, Walter King Yan Ho, Shaheen Begum, Guillermo Felipe López Sánchez

**Affiliations:** ^1^Core Curriculum Program, Department of Humanities and Social Sciences, Prince Mohammed Bin Fahd University, Al Khobor, Saudi Arabia; ^2^Faculty of Education, University of Macau, Taipa, China; ^3^Department of Commerce, Abeda Inamdar College, Pune University, Pune, India; ^4^Faculty of Health, Education, Medicine and Social Care, School of Medicine, Vision and Eye Research Institute, Anglia Ruskin University, Cambridge, United Kingdom

**Keywords:** need for approval, rumination, planfulness, striving for excellence, parental pressure

## Abstract

This study examined the relationships between perfectionism, self-esteem, and the will to win and the effects of gender and the level of achievement on these variables. A total of 318 adolescents in the age group of 12–19 years (*M* = 16.10 ± 1.01) completed the self-esteem questionnaire, the will-to-win questionnaire, and the perfectionism inventory. Interstate level (ISL) athletes obtained higher scores than interdistrict level (IDL) athletes on the following variables: self-esteem, the will to win, and four of the eight dimensions of perfectionism (i.e., concern over mistakes, the need for approval, organization, and planfulness). Further, male athletes obtained higher self-esteem and perfectionism (i.e., the need for approval and rumination) scores than female athletes. Self-esteem, the will to win, and the dimensions of perfectionism were positively and significantly interrelated. However, one dimension, namely, perceived parental pressure, was unrelated to any factor except striving for excellence. Further, the will to win, concern over mistakes, high standard for others, and planfulness were unrelated to striving for excellence. The results of the discriminant analysis revealed that there was no significant difference between ISL and IDL athletes (variance explained = 9.480%). Finally, using path analysis showed that Model 3 (perfectionism self-esteem will-to-win) has provided good model fit such as Bentler's comparative fit index (CFI) (0.987), Tucker-Lewis index (TLI) (0.876), normed fit index (NFI) (0.973), and the root mean square error of approximation (RMSEA) (0.097).

## Introduction

A characteristic feature of aspiring sportspersons is their pursuit of the attainment of high levels of performance. They tend to set high standards of performance for themselves, and this helps them accomplish their goals as a part of their pursuit to enhance their performance and skills. However, many athletes set unrealistic goals, and they believe that they need to accomplish them to achieve excellence (Burton and Naylor, 2002). Reportedly, individuals with perfectionistic cognitions often experience excessive anxiety (Lessin and Pardo, 2017). This is primarily the result of a discrepancy between the ideal self and the real self of an individual, and it can have detrimental effects on athletic performance. Several studies have found that a high level of motivation is an antecedent to optimal sports performance. In this regard, the cues that precede these behavioral patterns have been examined, and direction and intensity have been identified as key factors that play a role in action and effort (Iso-Ahola and St. Clair, 2000). The findings of several studies underscore the importance of motivation (Nicholls, 1989). These studies have tended to adopt goal orientation theory as their theoretical framework (Nicholls, 1989; Murcia et al., 2008). According to this theory, there are two types of goal orientations: (a) task orientation (i.e., emphasis on efforts and self-improvement) and (b) ego orientation (i.e., emphasis on outperforming others; Nicholls, 1989; Murcia et al., 2008). Therefore, this study aimed to examine the relationships between the different dimensions of perfectionism, self-esteem, and the will to win among adolescent athletes. This study also aimed to examine group differences (i.e., gender and achievement) in these variables.

### Perfectionism

Perfectionism is defined as “the personal highest standard which accepts excellence in a certain pursuit and rejects anything else” (Thompson, 1995, p. 1,015). Perfectionism is a multidimensional construct that encompasses multifaceted characteristics (Enns and Cox, 2002) and is prevalent among competitive athletes (Dunn et al., 2005). Sports psychologists have been interested in delineating the role that perfectionism plays in sports performance. Some consider it to be an adaptive trait that is shared by Olympic athletes, but others consider it to be a maladaptive trait that adversely affects performance. Therefore, researchers have been interested in resolving these contrasting perspectives (Flett and Hewitt, 2005). Perfectionism can be differentiated into two types, namely, personal standards and evaluative concerns. Personal standards perfectionism is striving for perfectionism that is rooted in personal standards and self-orientation (Dunkley et al., 2000; Blankstein et al., 2008). This dimension yields positive outcomes and affects and enhances performance. In contrast, evaluative concerns perfectionism is characterized by a preoccupation with the mistakes of an individual, self-doubt, and concerns about evaluations of an individual's performance by others. It results in negative outcomes such as anxiety, distress, and negative affect (Stoeber and Otto, 2006). Competitive athletes seek to attain excellence in whatever they pursue. Therefore, the achievement of excellence in a specific pursuit will enhance their sense of self-worth and, eventually, increase their self-esteem (Sonstroem et al., 1991). Slaney et al. (2001) found that normal perfectionism was more strongly correlated with self-esteem compared with neurotic perfectionism. Further, the relationship between normal perfectionism and self-esteem has been found to enhance self-competence and self-acceptance. In contrast, negative perfectionism exacerbates psychological problems such as depression, anxiety, and personality or interpersonal problems (Ghahramani et al., 2011). Based on these findings, the following hypotheses were formulated: there will be a significant difference in the level of perfectionism of (a) boys and girls and (b) those with different levels of achievement (*H*_*o*_*1*), and perfectionism will be positively correlated with self-esteem and the will to win (*H*_*o*_*2*).

### Will to Win

The will to win can be defined as a path toward the achievement of personal excellence and success in sports. It can also be defined as an intense urge to accomplish a voluntarily and consciously chosen goal. The will to win also entails tremendous inner strength and persistence toward a goal (e.g., life, sports, or any other endeavor). In one study, basketball players who possessed a stronger will to win were found to be more likely to win a game than their counterparts with a weaker will to win (Dorsey et al., 1980). However, of all the determinants of sports performance, desire and a will to win have been identified as the most influential factors. However, the interactionist approach to personality suggests that a will to win may vary across different sports and athletes with different levels of performance.

Singh and Reddy (2010) examined whether the will to win varies across different groups of athletes (*N* = 60; 15 short-distance runners, 15 long-distance runners, 15 jumpers, and 15 throwers). In the aforementioned study, the will to win was conceptualized as the intensity of the desire of an individual to defeat an opponent or exceed established standards of performance. The researchers found that long-distance runners had the strongest will to win, followed by throwers, short-distance runners, and jumpers. However, in a study that was conducted among 60 female athletes (15 short-distance runners, 15 long-distance runners, 15 jumpers, and 15 throwers), no significant group differences were found (Reddy et al., 2010). They concluded that differences between athletes are contingent on the intensity of their desire to defeat an opponent or exceed established standards of performance. Based on these findings, we proposed the following hypothesis: there will be a significant difference in the strength of the will of an individual to win between (a) boys and girls and (b) those with different levels of achievement (*Ho3*).

### Self-Esteem

Self-esteem refers to the extent to which an individual holds favorable attitudes toward oneself (Rosenberg, 1965). High levels of self-esteem yield positive outcomes (McAuley and Rudolph, 1995; McAuley et al., 2005) and make individuals feel good about themselves (Sonstroem and Morgan, 1989) by enhancing their well-being (Harter, 1993). Self-esteem is also considered to be a type of self-confidence that varies across different contexts (Weinberg and Gould, 2015); therefore, it informs players about evaluations of their own achievements. Several different motivational theories have been used to understand the role that domain-specific self-evaluations play in achievement-related behaviors. Domain-specific self-evaluations play a pivotal role in enhancing sports-centric motivational behavior (Harter, 1978; Nicholls, 1989). The study of Ahmed et al. (2020) highlighted that the adolescent athletes who participated at the national level showed higher levels of self-esteem and the will to win. They also reported a higher dispositional flow, especially in the factors of challenge skill balance, clear goals, and concentration on the task at hand. Besides, male athletes had significantly higher self-esteem and dispositional flow than female athletes. Furthermore, their study showed a significant positive relationship between self-esteem, will to win, and dispositional flow subscales. Several theories have posited that participation in sports and physical exercise and perceived competence are strongly related to intrinsic motivation (Bandura, 1986; Deci and Ryan, 2000). Several research findings suggest that self-esteem is closely associated with motivated behavior in physical activity settings (Goudas et al., 1994) and physical activity behaviors (Welk and Eklund, 2005). In an intervention study that was conducted among 11 to 15-year-old adolescents (*N* = 634), it was found that physical activity reinforces autonomous motives and enhances self-esteem. On the other hand, Tremblay et al. (2010) found that women and men who were more physically active had considerably higher levels of self-esteem than inactive students. High levels of physical activity were associated with higher levels of self-esteem, and this finding was valid, irrespective of the sex or age (range: elementary school to university students) of the participants (Frost and McKelvie, 2005). Another study found that individual sports athletes had higher levels of self-esteem and lower levels of anxiety than team sports athletes. Further, male athletes have been found to possess high levels of somatic anxiety and self-esteem than their female counterparts (Ichraf et al., 2013). Therefore, the following hypothesis was formulated: there will be significant differences in the self-esteem of (a) boys and girls and (b) those with different levels of achievement (*Ho4*).

### Sports in India, Theoretical Frameworks, and the Aim of This Study

In India, physical education (PE) and sports are not compulsory subjects. Therefore, only interested students choose to participate in games and sports. Further, the existing PE curriculum is unstructured and does not reward participation in sports. In addition, it fully depends on the school or the individual whether they want to pursue PE as a subject. Although PE is not compulsory for students, schools located in urban areas are more likely to pursue PE than those in rural and semirural areas (Ahmed et al., 2020). Sports and physical activity have a tremendous impact on the life of an adolescent and have been a major discussion of public health perspectives for a long time. Engaging in sports during adolescence is associated with an active lifestyle and enhances the general well-being. Although adolescence is a developmental period, it is associated with a certain degree of psychosocial vulnerability (Fuentes et al., 2020). Despite the numerous benefits of physical activity, adolescents tend to be less physically active and their participation in sports decreases with increasing age, which generally occurs between the ages of 15 and 16 years. These inconsistencies toward sports participation may be linked to several factors. However, if we relate this to the Indian context, some of these factors include low socio-economic status, lack of family support, peer support, and hardship to access sports facilities. Children rarely receive any encouragement and motivation from their parents to participate in sports. In Indian, parents usually prioritize “*study*” before participation in “*sport*.” Furthermore, family culture has also shown to be a strong evidence factor for facilitating propensities of individuals to play sports (Birchwood et al., 2008). Not all places in India have sufficient facilities (playground, stadium, equipment, etc.) to conduct sports activities; therefore, this obligates parents to take their children far away for sports practice. Often, this does not become possible for parents that have difficulties providing such support to their children, i.e., parents with low socio-economic status. Therefore, geographical accessibility (proximity) and affordability seem to be the major determinants of sports participation in adolescents (Eime et al., 2013). Adolescents spend a considerable amount of their free time with their peers as parental influence decreases (Ridao et al., 2021). Adolescence is not always the same in all cultural contexts, being differences between European and Anglo-Saxon countries compared to Eastern Societies (Yeung, 2021), especially for athletes (Sun et al., 2020). However, parents always remain as an important source of support to their children (Gallarin and Alonso-Arbiol, 2012). From this support, they perceive love and affection from their families, which enhances positivity for adjustment and competence (Martinez et al., 2021). Overall, parental practices based on warmth and involvement are related to adolescent competence and adjustment (Queiroz et al., 2020).

The study of Weiss and Amorose (2008) highlighted that concurrently students face major barriers (e.g., unsupportive parents and coaches, gender discrimination, and financial lacuna) that adversely affect their sports performance, growth, and prospects of success. The findings of this study, which examined the levels of perfectionism, self-esteem, and the will to win of adolescent student athletes, were expected to offer valuable information that addresses their need for improved performance. This study also examined gender differences and differences between athletes with different levels of achievement. Across all regions in India, the participation of women in sports appears to be a neglected issue. It is surprising that similar findings have also been reported in many other developed and developing countries. There has been a steep decline in the participation of adolescent girls (14–15 years) in sports (Pate et al., 2007; Bélanger et al., 2009). Therefore, this study, which adopted the competence motivational theory of Harter (1978, 1981) as a conceptual framework, aimed to offer developmentally appropriate statistics of these schools were primariding to the questionnaires were addressed, if these variables an activity ment, and per explanation for motivated behavior, performance, and achievement attrition within the domains of physical activity and sports across the different age groups.

Adolescents possess high levels of self-determination, tend to be drawn to challenges, discover their own strengths and weaknesses, and pursue goals and success (including mental toughness). In this regard, they seek psychological support and motivation from their peers, family members, and society at large. Nonetheless, the unsupportive and unstructured sports environment in India has had a negative effect on their performance, and it is an important issue that should be addressed. Therefore, the primary aim of this study was to examine group (i.e., gender and achievement) differences in perfectionism, self-esteem, and the will to win. Only a few studies have addressed these issues, and none of them were conducted in India. According to competence motivation theory, mastery experiences promote a sense of self-efficacy. Such efforts are also major contributors to desirable outcomes such as self-efficacy beliefs, autotelic experiences, emotional intelligence, enjoyment, and perceived competence. Children tend to continue engaging in an activity for a long period of time when they feel competent and derive enjoyment from their participation (Weiss et al., 2009). Additionally, social agents and contexts play a crucial role in sustaining achievement behaviors among adolescents (Weiss and Amorose, 2008). In summary, competence motivation theory lucidly delineates the role that social agents play in promoting positive attitudes among adolescents by enhancing the motivational levels of athletes and the enjoyment that they derive from participating in an activity (Weiss and Amorose, 2008). Reportedly, a decrease in such scholarship can adversely affect the levels of motivation, enjoyment, and future commitment to an activity of the athletes (Weiss et al., 2009). Therefore, based on a review of the literature and the aforementioned theoretical arguments regarding the participation of Indian youth in sports, this study aimed to determine their levels of perfectionism, self-esteem, and the will to win and examine group (i.e., gender, achievement) differences in these variables.

## Methods

### Participants

Using a stratified random sampling method, a total of 318 adolescent athletes were recruited from various higher secondary schools in Maharashtra, India. The sample consisted of 188 (47.6%) boys and 130 (52.4%) girls. Their ages ranged from 12 to 19 years (*M* = 16.10 ± 1.01). Further, they had participated in various interdistrict and interstate games and sports competitions. All the students were recruited from the Indian Council of Secondary Education (ICSE) schools. With regard to their levels of achievements, 143 (44.9%) of them had participated in interdistrict competitions, and 175 (55.1%) of them had participated in interstate competitions (Table 1).

**Table 1:** Demographic information.

**Level of achievement**			**Types of sports**			**Total**
			**Volleyball**	**Basketball**	**Football**	
Inter district level	Gender	Male	33	22	24	79
		Female	39	10	15	64
		Total	72	32	39	143
Inter state level	Gender	Male	24	44	41	109
		Female	27	25	14	66
		Total	51	69	55	175
Total	Gender	Male	57	66	65	188
		Female	66	35	29	130
		Total	123	101	94	318

### Ethical Considerations

This study was reviewed and approved by the India First Foundation, India. The participants were recruited from various schools, and their participation in this study was completely voluntary. Prior to data collection, the principal investigator of this study met the principals, teachers, and coaches of the school and informed them about the aims of this study. Written informed consent to participate in this study was provided by the athletes, their parents (for minors), and their coaches. All collected data were coded in order to maintain the confidentiality of participants. The student athletes were also informed about the study, and any doubts that they had while responding to the questionnaires were addressed. They were requested to answer all the items in the questionnaires, including the ones that required them to provide their personal information. Further, they were assured that their personal information and responses would be kept confidential.

### Procedure

Standardized assessments were used to collect data. Data were collected from the students of various ICSE schools. A substantial proportion of the data were also collected during the National Volleyball Tournament, which was held at the India First Foundation School in Karjat and Mumbai, India. Data collection was completed by the end of 2015. The athletes were recruited from ICSE schools. Therefore, only the English versions of the questionnaires were administered. The schools were located in Karjat and Mumbai. In these schools, English was the primary medium of instruction and examination. Therefore, the participants could complete these assessments in English.

The demographic characteristics of the participants are presented in Table 1.

### Measures

#### Self-Esteem

The 10-item Rosenberg Self-Esteem Questionnaire (Rosenberg, 1965) was used in this study. It consists of positively and negatively worded items that assess self-esteem, self-worth, and current attitudes toward oneself. Responses to the items are recorded on a 4-point Likert scale (Strongly Agree = 3, Agree = 2, Disagree = 1, and Strongly Disagree = 0). Items 3, 5, 8, 9, and 10 are reverse scored. Five items are positively worded (e.g., “On the whole, I am satisfied with myself,” “I feel that I have a number of good qualities”), and another five items assess negative self-evaluations (e.g., “At times, I think I am no good at all,” “I feel I do not have much to be proud of”). Total scores can range from 0 to 30. It has high reliability (0.80). Its internal consistency and test-retest reliability coefficients range from 0.77 to 0.88 and 0.82 to 0.85, respectively (Ahmed et al., 2017). In this study, its Cronbach's alpha was 0.90.

#### Will to Win

The 14-item will-to-win questionnaire, which has been developed by Pezer and Brown (1980), was used in this study. It is a sports-specific self-report measure that assesses the extent to which a person desires to achieve a given standard of excellence or defeat an opponent. This questionnaire consists of true–false items. Individual item scores (true = 0, false = 1) should be averaged to compute a total score. Scores that approximate 0 are indicative of a stronger will to win. Seven items are reverse scored (e.g., “I am more likely to swear when we're losing than when we're,” “I go into most games thinking we are going to win,” “During a game I sometimes feel sorry for opponents”), and seven items are positively worded (e.g., “I hate to lose,” “A team can be considered successful without winning,” “I don't mind when teammates give <100%”). In a pilot study that was conducted among 254 undergraduates who regularly participated in one of the seven sports, the test-retest (4 months) reliability of this scale was found to be 0.87, and the Kuder–Richardson reliability coefficient was 0.66. In this study, the Cronbach's alpha of this scale was 0.43.

#### Perfectionism

To assess the multidimensional facets of perfectionism, the perfectionism inventory (59 items) by Hill et al. (2004) was used. This inventory consists of eight dimensions: concern over mistakes (CM; e.g., “If I make mistakes, people might think less of me”), high standards for others (HSO; e.g., “I usually let people know when their work isn't up to my standards”), need for approval (NA; e.g., “I am over-sensitive to the comments of others”), organization (O; e.g., “I think things should be put away in their place”), perceived parental pressure (PPP; e.g., “My parents hold me to high standards”), planfulness (P; e.g., “I find myself planning many of my decisions”), rumination (R; e.g., “I spend a lot of time worrying about things I've done, or things I need to do”), and striving for excellence (SE; e.g., “My work needs to be perfect, in order for me to be satisfied”). Responses can be recorded on a 5-point scale (1 = Strongly Disagree, 5 = Strongly Agree). All the subscales have excellent internal consistency (range = 0.83–0.91) and test-retest (3–6 weeks) reliability (range = 0.71–0.91).

#### Data Analysis

Descriptive statistics (means, SDs, skewness, and kurtosis) are presented in Table 2. None of the skewness and kurtosis values exceeded the accepted limits (−2 to +2). This indicated that the assumption of normality was met (Table 2).

**Table 2:** Descriptive statistics, intercorrelations, and reliability.

**Sl. No**.	**Factors**	**1**	**2**	**3**	**4**	**5**	**6**	**7**	**8**	**9**	**10**
1	Self-esteem	1	0.488^**^	0.263^**^	0.497^**^	0.549^**^	0.448^**^	−0.028	0.346^**^	0.331^**^	0.126^*^
2	Will-to-Win		1	0.204^**^	0.399^**^	0.476^**^	0.351^**^	−0.100	0.224^**^	0.274^**^	−0.005
3	Concern over mistake			1	0.333^**^	0.435^**^	0.229^**^	−0.067	0.228^**^	0.334^**^	0.018
4	High standard for others				1	0.709^**^	0.593^**^	−0.017	0.472^**^	0.526^**^	0.116
5	Need for approval					1	0.560^**^	−0.071	0.415^**^	0.616^**^	0.126^*^
6	Organization						1	0.104	0.568^**^	0.427^**^	0.281^**^
7	Perceived parental pressure							1	0.047	0.075	0.659^**^
8	Planfullness								1	0.414^**^	0.111
9	Rumination									1	0.127^*^
10	Striving for excellence										1
	Alpha α	0.896	0.427	0.771	0.621	0.653	0.596	0.732	0.439	0.541	0.914
	Mean	30.24	9.94	17.05	14.94	16.11	15.72	15.10	15.09	14.91	15.10
	*SD*	7.27	2.49	2.40	3.08	2.75	2.81	2.73	2.80	3.20	2.87
	Skewness	−0.371	−0.407	−1.14	−0.781	−1.30	−0.781	−1.14	−0.695	−0.917	1.01
	Kurtosis	−1.07	0.202	1.86	0.432	2.59	0.770	1.61	0.322	0.769	1.33
	Minimum	13.00	2.00	6.00	4.00	4.00	6.00	4.00	5.00	4.00	5.00
	Maximum	40.00	15.00	20.00	20.00	20.00	20.00	20.00	20.00	20.00	20.00
Male (Mean)	30.84	9.86	28.88	27.44	30.03	30.39	30.22	26.12	27.67	26.00	
Male (Standard Deviation)	6.83	2.54	5.26	5.25	5.38	4.56	4.63	4.01	5.06	5.36	
Female (Mean)	29.37	10.04	28.32	26.58	28.86	29.66	29.58	25.39	26.11	25.18	
Female (Standard Deviation)	7.82	2.41	4.88	5.76	5.85	4.91	4.47	3.98	5.16	6.19	

*Error for Skewness = (0.137), Kurtosis = (0.273)*.

*1, self-esteem; 2, Will-to-Win; 3, concern over mistake; 4, high standard for others; 5, need for approval; 6, organization; 7, perceived parental pressure; 8, planfullness; 9, rumination; 10, striving for excellence*.

* *significant at 0.05 level of confidence*.

** *significant at 0.001 level of confidence*.

The demographic characteristics of the participants were examined by computing frequencies and percentages. With regard to the assessments, raw scores were computed in accordance with the instructions outlined in their manuals, and means and SDs were calculated. An independent-samples *t*-test was conducted to examine group (i.e., gender, achievement) differences. Discriminant function analysis was conducted to arrive at a combination of variables that predicts group membership (i.e., participants with different levels of achievement).

This study aimed to develop a model that includes all pertinent variables. Further, rigorous procedures were adopted to ensure that the model is applicable to both male and female athletes. Hence, path analysis was conducted to examine mediation effects, and the maximum likelihood estimation was used.

Model fit was examined by inspecting the results of the chi-squared test, the root mean square error of approximation (RMSEA), Bentler's comparative fit index (CFI), and Tucker-Lewis index (TLI). All the variables were treated as observed variables. This rendered path analysis similar to structural equation modeling (SEM).

By including fewer parameters in the model, we were able to enhance the parsimony of the model and better leverage our sample size. The data were analyzed using SPSS version 20.0. RMSEA values ≤0.08 are indicative of a reasonable fit. CFI and TLI values >0.95 are indicative of an excellent model fit (Kline, 2005).

The proportions of variance explained were ascertained by computing squared multiple correlations (*R*^2^). IBM SPSS version 20.0 was used to compute descriptive statistics and conduct correlational analyses, and AMOS version 20.0 was used to conduct SEM.

## Results

The data were analyzed using IBM SPSS (20.0). Descriptive statistics (i.e., means and SDs) were computed. An independent-samples *t*-test was used to examine gender differences and differences between groups with different levels of achievement. Zero-order correlations were computed to examine the relationships between the study variables. Discriminant function analysis was conducted to arrive at a combination of variables that predicts membership to the groups that differ in their levels of achievement. Subsequently, path analysis was conducted (AMOS 20.0). Mediation effects were tested using the maximum likelihood estimation. Model fit was assessed by inspecting the results of the chi-squared test, RMSEA, Bentler's CFI, and TLI. Since the reliability of the variables had already been established, they were treated as observed variables. This rendered path analysis identical to SEM; path analysis can be regarded as a special type of SEM in which only the observed variables are included in the causal model. In contrast, SEM is used to estimate latent variables that are founded upon observed variables. By including fewer parameters in the model, we were able to enhance the parsimony of the model and better leverage our sample size.

Means, SDs, Cronbach's alphas, and correlation coefficients for the study variables are presented in Table 2. The magnitudes of the intercorrelations among the variables ranged from weak to strong (−0.005 to 0.616). Moderate to strong positive correlations emerged between all variables except PPP, which was weakly and negatively correlated with all the factors. Further, the will to win was not correlated with SE.

### Path Analysis

**Figure 1:**
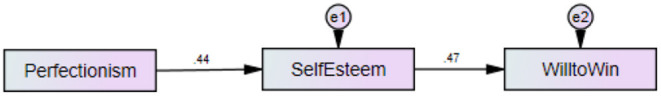
Path model depicting direct and indirect relationships between perfectionism, self-esteem, and the will to win.

The path model included three factors (i.e., self-esteem, will to win, and perfectionism). Using AMOS 20.0, SEM was undertaken to test the proposed structural relationships between the study variables. In order to find the best fitting model, three alternative models were evaluated. The fit indices of the alternative models are presented in Table 4. The hypothesized model (Model 3) was found to be an excellent fit for the data. The corresponding fit indices were as follows: CMIN/*df* = 3.988, *df* = 1, CFI = 0.979, TLI = 0.876, NFI = 0.973, RMSEA = 0.049. The parameter estimates indicated that all the direct path coefficients were significant and in the predicted direction. Self-esteem partially mediated the relationship between perfectionism and the will to win (Figure 1).

### Gender Differences

Multigroup analysis was conducted to examine gender differences in the path coefficients. Gender differences were examined by constraining the structural paths of the two models (i.e., for male and female athletes) to be equal.

**Figure 2:**
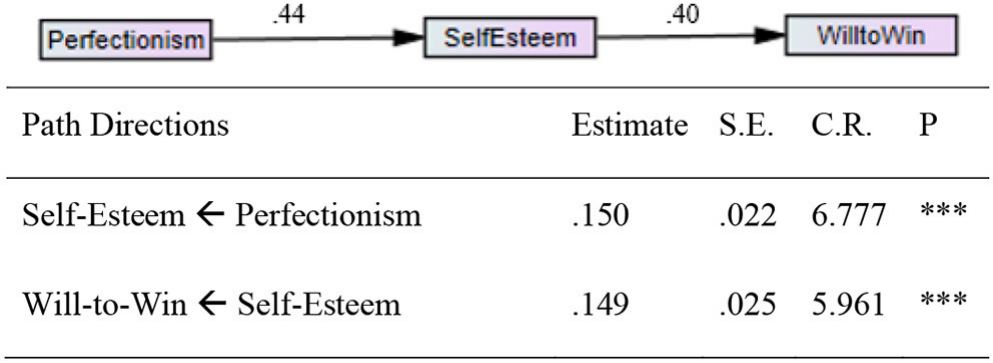
Path analysis of the latent construct using data obtained from male athletes.

**Figure 3:**
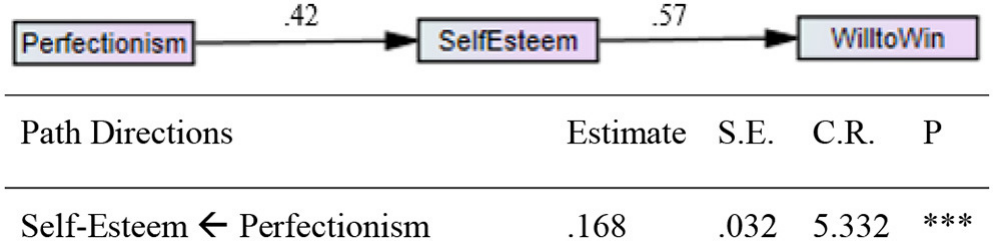
Path analysis of the latent construct using data obtained from female athletes.

An inspection of each path coefficient confirmed that the magnitude of all the associations was similar between male and female athletes. There was no significant gender difference. These results underscore the robustness of the meditation model (Figures 2, 3).

When compared to interdistrict level (IDL) athletes, interstate level (ISL) athletes obtained higher mean scores on the assessments that were used to measure self-esteem and the will to win. They also obtained higher scores on the following dimensions of perfectionism: CM, HSO, NA, O, PPP, P, and R. In contrast, IDL athletes obtained higher scores on the SE dimension. An independent-samples *t*-test was conducted to compare the mean scores that the two groups of athletes (i.e., IDL and ISL athletes) obtained on the assessments that were used to measure self-esteem, the will to win, and perfectionism. There was a significant difference in the self-esteem scores of the ISL (*M* = 31.14 ± 6.77) and IDL [*M* = 29.13 ± 7.73; *t*_(316)_ = 2.46, *p* < 0.05, two-tailed] athletes. The magnitude of the mean difference [*MD* = 2.00, 95% confidence interval (CI)] was large (η^2^ = 0.27). This suggests that the ISL athletes had a significantly stronger will to win than their IDL counterparts. There was a significant difference in the will-to-win scores of the ISL (*M* = 10.24 ± 2.32) and IDL [*M* = 9.56 ± 2.63; *t*_(316)_ = 2.43, *p* < 0.05] athletes. The magnitude of the mean difference (*MD* = 0.679, 95% CI) was large (η^2^= 0.27). This indicates that the ISL athletes had a significantly stronger will to win than the IDL athletes. There were significant differences in the following dimensions of perfectionism: CM [IDL athletes: M =, SD =; ISL athletes: M =, SD =; *t*_(*df*)_ =, *p* < 0.05], NA [IDL athletes: M =, SD =; ISL athletes: M =, SD =; *t*_(*df*)_ =, *p* < 0.05], O [IDL athletes: M =, SD =; ISL athletes: M =, SD =; *t*_(*df*)_ =, *p* < 0.05], and P [IDL athletes: M =, SD =; ISL athletes: M =, SD =; *t*_(*df*)_ =, *p* < 0.05]. The magnitude of the mean differences in CM (*MD* = 1.90, 95% CI; η^2^ = 0.37), NA (MD = −1.53, 95% CI; eta squared = 0.27), O (MD = −1.07, 95% CI; η^2^= 0.24), and P (MD = 1.79, 95% CI) was moderate (η^2^ = 0.20). This suggests that ISL athletes obtained significantly higher scores on CM, NA, O, and P. In contrast, differences in HSO, PP, R, and SE were not significant (Table 3).

**Table 3:** Gender-based descriptive statistics and differences on the variables using independent *t*-test.

**Factor**	**Gender**		***t*** **-test for Equality of Means**				**Eta square**
	**Male (Mean ±*SD*)**	**Female (Mean ±*SD*)**	***t***	**Sign. (*p*-value) (2-tailed)**	***MD***	***R***	
Self-esteem	(30.84 ± 6.83)	(29.37 ± 7.82)	1.77^*^	0.077	1.46	0.099	0.20
Will-to-Win	(9.86 ± 2.54)	(10.04 ± 2.41)	−0.630	0.529	−0.179	0.136	0.07
Concern over mistake	(28.88 ± 5.26)	(28.32 ± 4.88)	0.969	0.333	0.565	0.140	0.11
High Standard for others	(27.44 ± 5.25)	(26.58 ± 5.76)	1.37	0.171	0.856	0.142	0.15
Need for approval	(30.03 ± 5.38)	(28.86 ± 5.85)	1.82^*^	0.069	1.16	0.156	0.20
Organization	(30.39 ± 4.56)	(29.66 ± 4.91)	1.37	0.171	0.737	0.160	0.15
Perceived parental pressure	(30.22 ± 4.63)	(29.58 ± 4.47)	1.22	0.222	0.638	0.161	0.14
Planfullness	(26.12 ± 4.01)	(25.39 ± 3.98)	1.59	0.111	0.730	0.172	0.18
Rumination	(27.67 ± 5.06)	(26.11 ± 5.16)	2.67^*^	0.008	1.56	0.149	0.30
Striving for excellence	(26.00 ± 5.36)	(25.18 ± 6.19)	1.25	0.212	0.815	0.150	0.14

*Male N = 188, Female N = 130, and df = 316*.

* *significant at 0.05 level of confidence*.

**Table 4:** Measurement model.

**Regression paths**	**CMIN/DF**	**DF**	**CFI**	**TLI**	**NFI**	**RMSEA**
Model 1 (Self-esteem → Will to Win → Perfectionism)	43.884	1	0.703	0.782	0.708	0.367
Model 2 (Will to Win → Perfectionism → Self-esteem)	52.931	1	0.640	1.159	0.648	0.404
Model 3 (Perfectionism → Self-esteem → Will to Win)	3.988	1	0.979	0.876	0.973	0.049

When compared to female athletes, male athletes obtained higher self-esteem, NA, and R (i.e., dimensions of perfectionism) scores. In contrast, female athletes had a stronger will to win. An independent-samples *t*-test was conducted to compare the self-esteem, will-to-win, and perfectionism scores of male and female participants. There was a significant difference in the self-esteem scores of male (*M* = 30.84 ± 6.83) and female athletes [M = 29.37 ± 7.82; *t*_(316)_ = 1.77, *p* > 0.05, two-tailed]. The magnitude of the mean difference (*MD* = 1.46, 95% CI) was large (η^2^ = 0.20). This suggests that male athletes had significantly higher levels of self-esteem than female athletes. With regard to NA, there was a significant difference between male (*M* = 30.03 ± 5.38) and female athletes [*M* = 26.11 ± 5.16; *t*_(316)_ = 1.16, *p* > 0.05, two-tailed]. The magnitude of the mean difference (*MD* = 1.46, 95% CI) was large (η^2^ = 0.20). This indicates that male athletes had a significantly greater NA than female athletes did. Further, there was a significant difference in the R scores of male (*M* = 27.67 ± 5.06) and female athletes [*M* = 28.86 ± 5.85; *t*_(316)_ = 1.56, *p* < 0.05, two-tailed]. The magnitude of the mean difference (*MD* = 1.46, 95% CI) was large (η^2^ = 0.30). This suggests that male athletes ruminated to a significantly greater extent than female athletes did. Differences in the will to win [Males: 9.86 ± 2.54, Females: 10.04 ± 2.41, *t*_(316)_ = 0.630, *p* < 0.05, two-tailed], CM [Males: 28.88 ± 5.26, Females: 28.32 ± 4.88, *t*_(316)_ = 1.56, *p* < 0.05, two-tailed], and HSO [Males: 27.44 ± 5.25, Females: 26.58 ± 5.76, *t*_(316)_ = 1.37, *p* < 0.05, two-tailed] were significant. Differences in O [Males: 30.39 ± 4.56, Females: 29.66 ± 4.91, *t*_(316)_ = 1.37, *p* > 0.05, two-tailed], PPP [Males: 30.22 ± 4.63, Females: 29.58 ± 4.47, *t*_(316)_ = 1.22, *p* > 0.05, two-tailed], P [Males: 26.12 ± 4.01, Females: 25.39 ± 3.98, *t*_(316)_ = 1.56, *p* > 0.05, two-tailed], and SE [Males: 26.00 ± 5.36, Females: 25.18 ± 6.19, *t*_(316)_ = 1.25, *p* > 0.05, two-tailed] were not significant (Table 3).

Canonical discriminant functions were used in the analysis. Discriminant function analysis was conducted to arrive at a combination of variables that predicts membership to the two groups of participants who differed in their level of achievement. The results (Wilks' λ = 0.948) revealed that the model explained only 9.480% of the variance. The *p*-value was >0.05; this indicated that the model was not significant and that there was no significant difference between the two groups. Further, the group centroids were significantly different (distance: higher centroid = 0.170 [ISL], lower centroid = −0.245 [IDL]) (Table 5).

**Table 5:** Discriminant function analysis showing differences between levels of achievement in dimensions of all factors.

**Level of achievements**	**Eigenvalue**	**Wilks' lambda**	**Canonical correlation**	**Chi-square**	***df***	**Sig**.
Inter district level and inter state level	0.054	0.948	0.227	16.476	9	0.058

*Canonical discriminant functions were used in the analysis*.

## Discussion

The overarching aim of this study was to examine the relationships between the will to win, self-esteem, and perfectionism. Further, to examine these relationships using rigorous methods, path analysis was conducted. The analysis was conducted using the entire dataset as well as the stratified data (i.e., based on gender).

### Level of Achievement

Achievement was differentiated into two levels: IDL and ISL. Significant group differences in self-esteem and the will to win were found. Further, there was no significant difference in any of the dimensions of perfectionism. Group differences in self-esteem were significant. ISL athletes obtained higher scores than IDL athletes (Russell, 2001).

Self-esteem is defined as self-assessment and self-evaluation (Sonstroem and Morgan, 1989; McAuley and Rudolph, 1995; McAuley et al., 2005). People with high self-esteem hold favorable attitudes toward themselves (Rosenberg, 1965). In this study, higher self-esteem scores were obtained by ISL athletes (i.e., the group with a higher level of achievement). Therefore, this finding can be attributed to their level of achievement, playing status, and the level at which they had been competing. Athletes who participate in high-level competitions also have a better quality of life (Fox, 1998), and this in turn nurtures situation-specific self-confidence, which is often regarded as self-esteem (Weinberg and Gould, 2015). This strongly informs how players evaluate their own achievements. An increase in the perceived physical value of a person also enhances self-esteem (Biddle, 1995) and goal-orientation (Duda, 1989). Motivation is a strong antecedent of achievement-related behavior, especially in relation to sports, and it plays a crucial role in cultivating such behaviors (Harter, 1978; Deci and Ryan, 1985; Nicholls, 1989). Therefore, this may be another reason why ISL athletes obtained higher self-esteem scores than IDL athletes.

Further, group differences in the will to win (*t* = 2.43, *p* < 0.015) were significant; ISL athletes obtained higher mean scores than IDL athletes (Russell, 2001). This finding is also attributable to their level of achievement, playing status, and the level at which they had been competing. Isolating the factors that are important determinants of participation in high-level competitions is a complex task. However, the will to win is considered to be one of the strongest determinants of achievement-related behaviors among athletes. The will to win is defined as an intense desire to accomplish something, achieve personal excellence, and succeed at sports. Moreover, the achievement of excellence is indubitably a product of substantial commitment, motivation, and willpower. They nurture a sense of internal strength and persistence toward the goals of an individual across different life domains (e.g., sports). Studies have also found that, once athletes begin to play in high-level competitions, they learn to cope with fatigue and pain, develop strategies to achieve their goals, and stay motivated. All these factors are prerequisites for winning a competition (Durand-Bush and Salmela, 2001). Notably, high levels of commitment can result in burnout in individuals with perfectionist traits. Therefore, this occurrence imbalances individual's emotion. The higher scores that were obtained by ISL athletes are consistent with this conceptualization. One study found that basketball teams with a stronger will to win were more likely to win a game than their weak-willed counterparts (Dorsey et al., 1980). However, of all the determinants of sports performance, desire and the will to win have been listed as the most influential factors. However, the interactionist approach to personality suggests that the will to win may vary across different sports and athletes with different levels of performance. Additionally, in India, sports are not as advanced in developing countries (e.g., infrastructure) as in other developed countries, and athletes often face various impediments that hinder their participation in sports. All aspiring athletes seek to excel at their chosen sport. Young athletes often try to convince their parents to allow them to participate in future competitions by demonstrating good performance. When they perform well, they gain popularity among their peers and within their communities, and they also aim to participate in high-level competitions. Therefore, this may be one of the reasons why ISL athletes reported a stronger will to win. Further, ISL athletes obtained higher means on the CM, NA, and O dimensions of perfectionism. The relationship between perfectionism and sports performance has always puzzled researchers.

The CM dimension of perfectionism is defined as a tendency to react negatively to the errors of an athlete. Athletes are quite conscious about their reputation, and they often equate mistakes with failure and believe that making any mistake will adversely affect their prestige (Frost et al., 1990). However, CM is linked to various factors such as parental pressure, coach pressure, high expectations from peers, and pressure from the federation/club/authorities of an athlete. Studies have found that authoritarian parenting is highly correlated with unhealthy perfectionist orientations among sportspersons (Gotwals et al., 2010). This may be one of the reasons why this cohort obtained higher mean scores on this factor.

When compared with IDL athletes, ISL athletes obtained higher scores on the NA dimension. Human beings possess an inquisitive and ambitious behavior when combining the thoughts and behavior resulting from high outstanding expectations and performances. Such behaviors are characteristic features of elite athletes. They are also more task-oriented and focused on excelling at their chosen sport. Blaney and Kutcher (1991) have propounded that NA is more strongly linked to sociotropy than to dependency. In other words, elite athletes are sensitive to appreciation, criticism, and support (Blaney and Kutcher, 1991). NA is defined as the tendency to seek validation from others and be sensitive to criticism.

Organization is defined as the tendency to be disciplined, be punctual, and adhere to a plan or schedule to enhance the athletic performance of an individual. Wanting to excel at sports is regarded as a healthy and positive type of perfectionistic goal (Stoeber and Otto, 2006) that is strongly influenced by parenting style (Speirs Neumeister, 2004). Indian adolescents try to meet parental expectations and demands by enhancing their performance (Stoeber and Otto, 2006). Therefore, ISL athletes may have obtained higher O scores. An authoritative parenting style has been linked to both positive and negative outcomes (Mouratidis and Michou, 2011). Continuous successes enhance motivation, but failures result in punishment and frustration (Deci and Ryan, 2012). Therefore, further research is needed to better understand this phenomenon and identify the factors that influence the development of organizational tendencies in young athletes.

There were significant gender differences in R. Indian adolescents spend more time on academics than on other extracurricular activities. This is also what parents expect and appreciate. Therefore, it is difficult for them to get parental permission to participate in sports/tournaments. This may be a reason why male athletes obtained higher R scores than female athletes. R is defined as the tendency to obsessively worry about past errors, less-than-perfect performances, and future mistakes. Adolescents may feel anxious about losing a tournament because it can adversely affect the perceptions of their parents about them and their likelihood of being permitted to participate in sports/tournaments in the future. Egan et al. (2013) found that there is a significant relationship between R and CM. In India, the rate of participation in sports is higher among men than among women. Therefore, male athletes may have obtained higher R scores than their female counterparts. Adolescence is a developmental period that is characterized by positivity, vitality, and creativity. They wish to excel at the things that they pursue in order to win the approval of their families and friends. In this regard, parental criticism is a crucial predictor of social anxiety (Nolen-Hoeksema, 2000) and depression (Kocovski et al., 2005; Rukmini et al., 2014). Further, Kocovski and Rector (2007) found that failure is a strong predictor of depressive symptoms. Rumination has also been linked to perfectionism and failure (Harris et al., 2008). Therefore, further research is needed to address these ambivalences in the literature.

### Strengths and Limitations

As a strength, this study provides evidence on the relationships between the different dimensions of perfectionism, self-esteem, and the will to win among adolescent athletes. The study also helped us to comprehend the underpinning attributes that herald male athletes to obtain higher self-esteem and perfectionism than their female counterparts. Striving for excellence has remained a pivotal aspect to aspiring athletes for enhancing their performance. Besides, *parental support* significantly contributes to the success of the children in their endeavors. On the contrary, *parental pressure* is also detrimental to the mental health and motivation of the children. Therefore, in this context, the present study provided details on how perceived parental pressure is associated with striving for excellence and leads toward a decline in their performance. This research has the potential to help PE teachers and sports coaches to allocate strategies for adolescent athletes to obtain excellence in their performance while perceiving parental pressures. Further, this resulted in restricting adolescents' progress in athletic endeavor. Despite the strengths mentioned earlier, this study has several limitations. First, the sample used for analysis is gender-unbalanced (female 52.4%; male 47.6%). In the future, more responses from male athletes should be garnered for analysis. Further, low generalizability of the results has also been presumed in this study to be due to the few participants per sport. Therefore, this study warrants future studies with more participants in each sports group. Besides, this study has invited participants (interdistrict and interstate games) from Maharashtra (state) only, which is considered a limitation. To get a true scenario of the relationships between the different dimensions of perfectionism, self-esteem, and the will to win among adolescent athletes, a study recruiting athletes from different parts of India will enhance its generalizability.

## Conclusion

In sum, the present findings delineate the relationships between the three latent variables (i.e., the will to win, self-esteem, and perfectionism). The present findings also specify the variables that differed as a function of gender and the level of achievement. Importantly, the results underscore the validity of the structural model, which was found to be an excellent fit for the data and offer insights into the effects of gender on this model.

## Data Availability Statement

The original contributions presented in the study are included in the article/Supplementary Material, further inquiries can be directed to the corresponding author/s.

## Ethics Statement

Permission to conduct this study was obtained from the India First Foundation School. Written informed consent to participate in this study was provided by the athletes, and their coaches.

## Author Contributions

MA: study design, data collection, data analysis, and drafting of the manuscript. SB: data collection, data entry, and drafting of the manuscript. GL and WY: drafting of the manuscript. All authors contributed to the article and approved the submitted version.

## Conflict of Interest

The authors declare that the research was conducted in the absence of any commercial or financial relationships that could be construed as a potential conflict of interest.

## Publisher's Note

All claims expressed in this article are solely those of the authors and do not necessarily represent those of their affiliated organizations, or those of the publisher, the editors and the reviewers. Any product that may be evaluated in this article, or claim that may be made by its manufacturer, is not guaranteed or endorsed by the publisher.
